# New Dammarane Triterpenoids, Caffruones A–D, from the Cherries of *Coffea arabica*

**DOI:** 10.1007/s13659-018-0181-y

**Published:** 2018-08-20

**Authors:** Xia Wang, Xing-Rong Peng, Jing Lu, Gui-Lin Hu, Ming-Hua Qiu

**Affiliations:** 1 State Key Laboratory of Phytochemistry and Plant Resources in West China, Kunming Institute of Botany, Chinese Academy of Sciences, Kunming, 650201 People’s Republic of China; 2 University of the Chinese Academy of Sciences, Beijing, 100049 People’s Republic of China

**Keywords:** *Coffea arabica*, Cherries, Triterpenoids, Structural elucidation

## Abstract

**Abstract:**

In present study, four new dammarane-type triterpenoids, namely caffruones A–D (**1**–**4**), were isolated from the cherries of *Coffea arabica*. Their structures were elucidated by extensive spectroscopic analysis including 1D, 2D NMR (HSQC, HMBC, ^1^H–^1^H COSY, and ROESY), HRMS and IR spectra. This is the first time that tetracyclic triterpenes have been reported in genus *Coffea*.

**Graphical Abstract:**

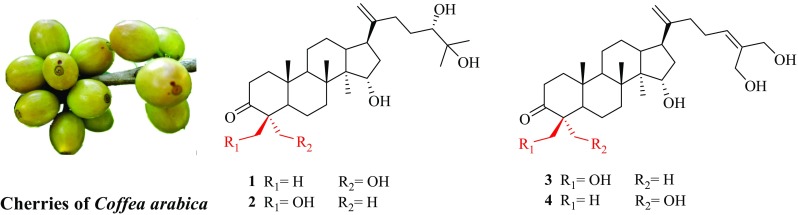

**Electronic supplementary material:**

The online version of this article (10.1007/s13659-018-0181-y) contains supplementary material, which is available to authorized users.

## Introduction

**Fig. 1:**
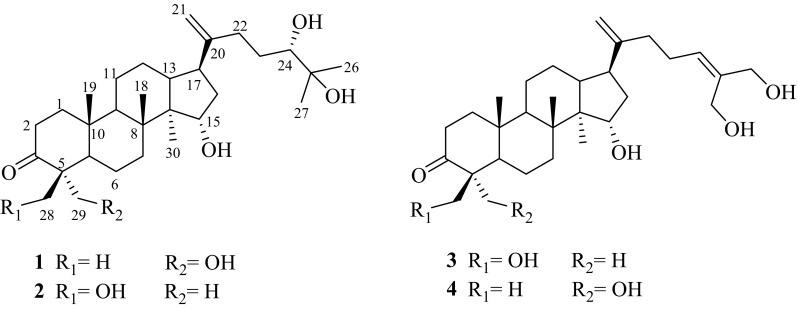
Structures of caffruones A–D (**1**–**4**) isolated from the cherries of *Coffea arabica*

*Coffea arabica* L. (Rubiaceae) is the most economic significant species in the coffee trade, occupying 69% of the world’s coffee production [1]. Yunnan province is the main cultivation base of coffee in China, and the cultivated species is almost *C. arabica*. In 2016, the total coffee production of Yunnan province was nearly 140 thousand tons [2]. Research found that coffee consumption has a variety of beneficial effects on human health, like anti-Alzheimer’s disease [3], anti-diabetes [4] and anti-cancers [5]. Previous phytochemical investigations on green and roasted coffee beans have resulted in the isolation of caffeine, trigonelline, chlorogenic acids, phenolic acids and a series of *ent*-kaurane diterpenoids [6–8]. All of them contributed to the healthy functions of coffee brews [9, 10]. However, to our best knowledge, there is no research has been focused on the chemical constituents of the coffee cherries. Therefore, as part of our systematic phytochemical investigation on *C. arabica* cultivated in Yunnan province, we isolated four new dammarane triterpenoids (**1**–**4**) (Fig. 1) from the coffee cherries. This is the first time that dammarane triterpenoids have been reported in genus *Coffea*. Herein, the isolation and structural elucidation of all isolates were described.

## Results and Discussion

The methanol extract of air-dried coffee cherries was subjected to multiple chromatographic steps, involving silica gel column chromatography, preparative thin-layer chromatography (P-TLC) and semi-preparative HPLC to yield four dammarane triterpenoids (**1**–**4**).

Caffruone A (**1**) was isolated as white amorphous powder and the molecular formula C_30_H_50_O_5_ was deduced from its [M + Na]^+^ ion peak at *m*/*z* 513.3551 (calcd 513.3550), with six degrees of unsaturation. The ^1^H NMR spectrum (Table 1) exhibited the proton signals for six singlet methyls (*δ*_H_ 0.95, 1.01, 1.06, 1.12, 1.17, 1.23), one olefinic methylene [(*δ*_H_ 4.78, s, H-21a), (*δ*_H_ 4.74, d, *J* = 1.8 Hz, H-21b)], one oxygenated methylene [(*δ*_H_ 3.43, 3.38), each 1H, *J* = 11.3 Hz, H-28a and H-28b] and two oxygenated methines [(*δ*_H_ 3.38 (dd, *J* = 10.6 and 1.9 Hz, H-24), *δ*_H_ 4.28 (t, *J* = 8.6 Hz, H-15)]. The ^13^C-DEPT NMR spectra (Table 2) suggested that **1** was a triterpenoid derivative with a total 30 carbons, consisiting of six methyls, eleven methylenes (one oxygenated and one olefinic), six methines (two oxygenated) and seven quaternary carbons (one oxygenated, one olefinic and one ketone carbonyl). Apart from a double bond and a carbonyl groups, the remaining elements of unsaturation degrees contributed to four rings, therefore, **1** should be a tetracyclic triterpenoid.

**Table 1:** ^1^H NMR spectroscopic data of compounds **1**–**4** [*δ* in ppm, *J* in Hz]

Position	**1** ^a^	**2** ^a^	**3** ^a^	**4** ^a^
1	2.01 (m), 1.40 (m)	1.93 (m), 1.57 (m)	1.93 (m), 1.57(m)	2.01 (m), 1.40 (m)
2	2.62 (m), 2.30 (m)	2.59 (m), 2.38 (m)	2.59 (m), 2.38 (m)	2.62 (m), 1.70 (m)
3	–	–	–	–
4	–	–	–	–
5	1.67 (m)	1.63 (m)	1.64 (m)	1.67 (m)
6	1.42 (m), 1.57 (m)	1.62 (m), 1.50 (m)	1.63 (m), 1.58 (m)	1.56 (m), 1.41 (m)
7	1.70 (m), 1.56 (m)	1.60 (m), 1.47 (m)	1.59 (m), 1.48 (m)	2.31 (m), 1.57 (m)
8	–	–	–	–
9	1.42 (m)	1.38 (m)	1.39 (m)	1.43 (m)
10	–	–	–	–
11	1.55 (m), 1.24 (m)	1.50 (m), 1.40 (m)	1.51 (m), 1.19 (m)	1.56 (m), 1.27 (m)
12	1.59 (m), 1.70 (m)	1.71 (m), 1.58 (m)	1.57 (m), 1.16 (m)	1.57 (m), 1.17 (m)
13	1.71 (m)	1.70 (m)	1.67 (m)	1.68 (m)
14	–	–	–	–
15	4.28 (t, 8.6)	4.25 (t, 8.6)	4.25 (d, 8.5)	4.26 (t, 8.7)
16	1.89 (m), 1.68 (m)	1.86 (m), 1.68 (m)	1.84 (m), 1.67 (m)	1.85 (m), 1.68 (m)
17	2.25 (m)	2.25 (m)	2.24 (m)	2.24 (m)
18	1.12 (s)	1.07 (s)	1.06 (s)	1.12 (s)
19	1.06 (s)	0.90 (s)	0.90 (s)	1.07 (s)
20	–	–	–	–
21	4.78 (s), 4.74 (d, 1.8)	4.78 (s), 4.74 (s-like)4.76 (d, 15.5)	4.75 (overlapped)	4.78 (s), 4.74 (s-like)
22	2.24 (m), 2.01 (m)	2.25 (m), 2.00 (m)	2.24 (m), 2.01 (m)	2.01 (m), 2.25 (m)
23	1.62 (m), 1.45 (m)	1.63 (m), 1.44 (m)	2.24 (m), 2.01 (m)	2.25 (m), 2.01 (m)
24	3.38 (dd, 10.6, 1.9)	3.38 (dd, 10.5, 2.0)	5.60 (t, 7.2)	5.56 (t, 7.2)
25	–	–	–	–
26	1.23 (s)	1.22 (s)	4.31 (s)	4.33 (s)
27	1.17 (s)	1.17 (s)	4.21 (s)	4.22 (s)
28	3.43 (d, 11.3), 3.64 (d, 11.3)	1.27 (s)	1.27 (s)	3.65 (d, 11.3), 3.42 (d, 11.3)
29	1.01 (s)	3.98 (d, 11.2), 3.45 (d, 11.2)	3.98 (d, 11.2), 3.45 (d, 11.2)	1.07 (s)
30	0.95 (s)	0.96 (s)	0.96 (s)	0.96 (s)

^a^Data were measured at 600 MHz in CDCl_3_

**Table 2:** ^13^C NMR spectroscopic data of compounds **1**–**4** [*δ* in ppm]

Position	**1** ^a^	**2** ^a^	**3** ^a^	**4** ^a^
1	39.6 (t)	39.7 (t)	39.7 (t)	39.6 (t)
2	35.3 (t)	34.2 (t)	34.2 (t)	35.2 (t)
3	219.0 (s)	221.3 (s)	221.4 (s)	219.0 (s)
4	52.4 (s)	50.8 (s)	50.8 (s)	52.5 (s)
5	49.3 (d)	55.5 (d)	55.5 (d)	49.3 (d)
6	19.0 (t)	19.0 (t)	19.0 (t)	19.0 (t)
7	35.4 (t)	35.4 (t)	35.5 (t)	35.5 (t)
8	40.7 (s)	40.5 (s)	40.5 (s)	40.5 (s)
9	50.5 (d)	50.5 (d)	50.4 (d)	50.5 (d)
10	36.8 (s)	36.6 (s)	36.6 (s)	36.6 (s)
11	21.6 (t)	22.1 (t)	22.2 (t)	22.2 (t)
12	24.7 (t)	24.8 (t)	24.8 (t)	24.8 (t)
13	43.6 (d)	43.6 (d)	43.4 (d)	43.4 (d)
14	50.4 (s)	50.4 (s)	50.3 (s)	50.4 (s)
15	73.8 (d)	73.8 (d)	73.8 (d)	73.8 (d)
16	38.6 (t)	38.6 (t)	38.4 (t)	38.5 (t)
17	45.5 (d)	45.5 (d)	45.2 (d)	45.2 (d)
18	15.6 (q)	15.1 (q)	15.0 (q)	15.6 (q)
19	16.1 (q)	17.2 (q)	17.2 (q)	16.0 (q)
20	151.6 (s)	151.5 (s)	150.7 (s)	150.8 (s)
21	108.5 (t)	108.4 (t)	108.7 (t)	108.7 (t)
22	31.2 (t)	31.1 (t)	33.8 (t)	33.8 (t)
23	29.9 (t)	29.9 (t)	26.1 (t)	26.0 (t)
24	78.1 (d)	78.1 (d)	130.4 (d)	130.5 (d)
25	73.1 (s)	73.2 (s)	137.3 (s)	137.2 (s)
26	26.6 (q)	26.5 (q)	60.0 (t)	60.0 (t)
27	23.2 (q)	23.1 (q)	67.4 (t)	64.7 (t)
28	66.8 (t)	22.0 (q)	22.1 (q)	65.7 (t)
29	16.6 (q)	65.7 (t)	65.7 (t)	16.5 (q)
30	9.0 (q)	8.9 (q)	8.9 (q)	9.0 (q)

^a^Data were measured at 150 MHz in CDCl_3_

**Fig. 2:**
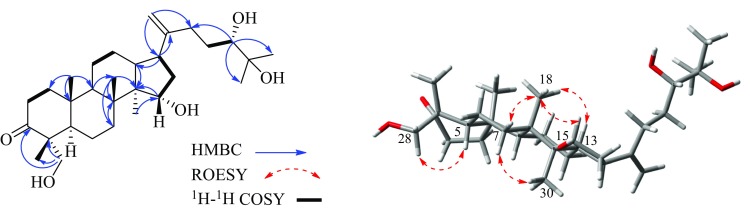
The key HMBC, ROESY and ^1^H-^1^H COSY correlations of compound **1**

Furthermore, the key HMBC correlations (Fig. 2) from H_3_-18 (*δ*_H_ 1.12) to C-7 (*δ*_C_ 35.4), C-14 (*δ*_C_ 50.4) and C-8 (*δ*_C_ 40.7), from H_2_-7 (*δ*_H_ 1.70, 1.56) to C-8 and C-18 (*δ*_C_ 15.6), from *δ*_H_ 1.43 (H-9) to C-18, and from H_3_-19 (*δ*_H_ 1.43) to C-9 (*δ*_C_ 50.5), C-10 (*δ*_C_ 36.8) and C-1 (*δ*_C_ 39.6), demonstrated that C-8 and C-10 in **1** were substituted by methyls (Fig. 2). Moreover, the ROESY correlations of *δ*_H_ 1.70 (H-7a)/*δ*_H_ 0.96 (H_3_-30, *α*-oriented), *δ*_H_ 1.56 (H-7b)/*δ*_H_ 1.12 (H_3_-18)/*δ*_H_ 1.71 (H-13) and *δ*_H_ 2.25 (H-17)/*δ*_H_ 0.96 (H_3_-30) confirmed that H_3_-18, H-13 were *β*-oriented and H-17 was *α*-oriented in **1** (Fig. 2). The evidence described above enabled the establishment of dammarane triterpene skeleton of **1** [11, 12].

Aforementioned information suggested that the structure of **1** were closely resembled to 24,25-dihydroxy-dammar-20-en-3-one [13], except that a methyl and an aliphatic methylene in the latter were replaced by an oxygented methylene (*δ*_C_ 66.8) and an oxygenated methine (*δ*_C_ 73.8) in **1**, respectively. The HMBC correlations from the methylene protons (*δ*_H_ 3.43, 3.64, H_2_-28) to C-3 (*δ*_C_ 219.0), C-4 (*δ*_C_ 52.4) and C-29 (*δ*_C_ 16.6), together with the ROESY correlation of H_2_-28/H-5 indicated that C-28 in **1** was connected a hydroxyl group (Fig. 2). Meanwhile, the HMBC correlations from H_3_-30 (*δ*_H_ 0.95) to the methine carbon (*δ*_C_ 73.8, C-15) and ^1^H-^1^H COSY correlations between *δ*_H_ 1.89, 1.68 (H_2_-16)/*δ*_H_ 4.28 (H-15) confirmed a hydroxyl group was located at C-15 in **1**.

Attempts to determine the absolute configuration of the 24,25-diol moiety in **1** by X-ray crystallography, Snatzke’s method and modified Mosher’s method were failed. However, on the analysis of the literatures available [14–17], the ^1^H and ^13^C NMR chemical shifts for two stereochemical configuration at C-24 of triterpenes with 24,25-diol moeity were distinct different. The 24*S* and 24*R* epimers of 24,25-dihydroxytiruall-7-en-3-one exhibited the chemical shifts of *δ* 3.32/78.6 (24*S*) and *δ* 3.29/79.5 (24*R*) [14], respectively. Thus, the chemical signals of **1** were *δ* 3.38/78.1 (H/C-24) indicated that the absolute configuration of C-24 in **1** was *S*. Moreover, This conclusion was surpported by the 1D NMR data of the related compound (24*S*)-24,25-dihydroxy-dammar-20-en-3-one [*δ* 3.39/78.3 (H/C-24)] [15]. Additionally, the OH-15 in **1** was assigned to be *α*-oriented by the ROESY correlation of H_3_-18/H-15 (Fig. 2). Therefore, the structure of **1** was established as (24*S*)-15*α*,24,25,28*α*-tetrahydroxy-dammar-20-en-3-one.

Caffruone B (**2**), obtained as white amorphous powder, was assigned the molecular formula C_30_H_50_O_5_ by HRESIMS, as the same as **1**. Moreover, its ^1^H and ^13^C NMR spectra (Tables 1, 2) were nearly superimposable with those of **1**, which indicated that they had the same planer structure. However, detailed analysis of the 2D NMR spectra showed that the main difference between **1** and **2** were present in their ROESY spectra. The oxygenated methylene protons (*δ*_H_ 3.98, 3.45, H_2_-29) showed correlation of *β*-oriented methyl protons H_3_-19 (*δ*_H_ 0.90) in **2**, rather than H-5*α* in **1**, suggesting that C-29 was the oxygenated methylene in **2**, not C-28 in **1**. Meanwhile, the ROESY correlation of H_3_-18 (*δ*_H_ 1.07)/H-15 (*δ*_H_ 4.25) proved the 15-OH to be *α*-oriented (supporting infomation). Similarly, the absolute configuration of C-24 in **2** was determined as *S* due to the same chemical shifts of C-24 with **1**. These results indicated that **1** and **2** were a pair of epimers. Therefore, **2** was elucidated as (24*S*)-15*α*,24,25,29*β*-tetrahydroxy-dammar-20-en-3-one.

Caffruone C (**3**) was isolated as white amorphous powder. Its molecular formula, C_30_H_48_O_5_, determined from the [M + Na]^+^ peak at *m*/*z* 511.3396 (calcd for 511.3394) in the HRESIMS spectrum, suggesting seven degrees of unsaturation. The ^1^H NMR spectrum (Table 1) showed the signals for four singlet methyls [*δ*_H_ 0.90, 0.96, 1.06, 1.27], two olefinic [*δ*_H_ 4.76 (overlapped, H_2_-21); *δ*_H_ 5.60 (t, *J* = 7.2 Hz, H-24)], three oxgenated methylenes [(*δ*_H_ 3.98, 3.45, each 1H, *J* = 11.2 Hz, H-29a and H-29b), *δ*_H_ 4.31 (s, H_2_-26), *δ*_H_ 4.21 (s, H_2_-27)], and one oxgenated methine [*δ*_H_ 4.25 (t, *J* = 8.5 Hz, H-15)]. The ^13^C-DEPT NMR spectra (Table 2) suggested that **3** was also a triterpenoid derivative with a total of 30 carbons, assigning to four methyls, thirteen methylenes (three oxgenated and one olefinic), six methines (one oxygenated and one olefinic), and seven quaternary carbons (two olefinic and one ketone carbonyl). Aparting the three degrees ocuppied by two double bonds and a carbonyl, the remaining degrees attributed to a tetracyclic system.

Similar with **1**, the key HMBC correaltions from H_3_-18 (*δ*_H_ 1.06) to *δ*_C_ 50.4 (C-9), *δ*_C_ 35.5 (C-7) and *δ*_C_ 40.5 (C-8), from H_3_-19 (*δ*_H_ 0.90) to *δ*_C_ 36.6 (C-10), 39.7 (C-1), *δ*_C_ 50.4 (C-9) and *δ*_C_ 55.5 (C-5), together with the ROESY correlations of *δ*_H_ 1.59 (H-7a)/*δ*_H_ 0.96 (H_3_-30), *δ*_H_ 1.48 (H-7b)/*δ*_H_ 1.06 (H_3_-18)/*δ*_H_ 1.67 (H-13) confirmed that **3** was also a dammarane triterpenoid.

**Fig. 3:**
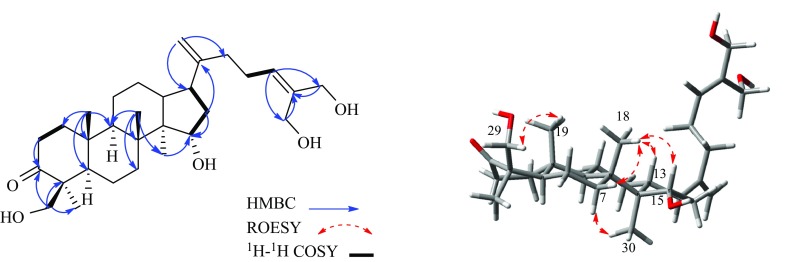
The key HMBC, ROESY and ^1^H–^1^H COSY correlations of compound **3**

Based on its 1D and 2D NMR data, **3** was assigned as a 3-oxo-dammara-20,24-dien-26-ol triterpenoid along with three additonal hydroxy groups [18]. The ^1^H-^1^H COSY correlations of *δ*_H_ 4.25 (H-15)/*δ*_H_ 1.84, 1.67 (H_2_-16) and HMBC correaltion from H-15 to *δ*_C_ 8.9 (C-30) comfired that C-15 in **3** was an oxygenated methine. In the HMBC spectrum, the correlations from the oxygenated methylene protons *δ*_H_ 4.21 (H_2_-27) to *δ*_C_ 60.0 (C-26) and *δ*_C_ 137.3 (C-25) and from the olefinic proton *δ*_H_ 5.60 (H-24) to *δ*_C_ 67.4 (C-27) deduced that C-27 in **3** was substituted by a hydroxy group. Moreover, the ROESY correlations of *δ*_H_ 3.98, 3.45 (H_2_-29)/*δ*_H_ 0.90 (H_3_-19) and HMBC correlations from H_2_-29 to *δ*_C_ 22.1 (C-28), *δ*_C_ 50.8 (C-4) and *δ*_C_ 221.4 (C-3) demonstrated that C-29 in **3** was also substituted by a hydroxy group (Fig. 3). Furthermore, the relative configuration of OH-15 in **3** was elucidated as α-oriented by the ROESY correlation of H-15 (*δ*_H_ 4.25)/H_3_-18 (*δ*_H_ 1.06). Thus, these spectral data established **3** as 15*α*,26,27,29*β*-tetrahydroxy-dammar-20,24-dien-3-one.

Caffruone D (**4**) was obtained as white amorphous powder and possessed the same molecular formula of C_30_H_48_O_5_ as **3**, as determined by HRESIMS data. The 1D NMR data (Tables 1, 2) of **3** were also observed in **4**, which indicated that they had the same planar structure. Similarly, the key ROESYcorrelations of an oxygenated methylene protons *δ*_H_ 3.62, 3.42 (H_2_-28) with *δ*_H_ 1.43 (H-5) demonstrated that C-28 was oxygenated methylene in **3**, not C-29 in **4**. Moreover, the ROESY correlation of *δ*_H_ 4.26 (H-15)/*δ*_H_ 1.12 (H_3_-18) indicated that the OH-15 was *α*-oriented (supporting information). These evidence suggested that **3** and **4** was also a pair of epimers. Therefore, the structure of **4** was characterized as 15*α*,26,27,28*α*-tetrahydroxy-dammar-20,24-dien-3-one.

## Experimental

### General Experimental Procedures

Ultraviolet spectra were measured by UV-2401 PC spectrophotometers (Shimadzu, Japan). A Bruker Tensor-27 instrument (Bruker, German) was used for recording infrared spectra by using KBr pellets. A Jasco P-1020 polarimeter (Jasco, Japan) was used to obtain optical rotations and HREIMS data were measured by an API QSTAR Pulsar spectrometer (Waters, UK). The Bruker DRX-600 instruments (Bruker, Zurich, Switzerland) were used to detect 1D and 2D NMR spectra with TMS as internal standard for chemical shifts. Semi-preparative HPLC was performed on an Agilent HP1100 or 1260 series instrument with a UV L-2400 detector (Agilent, USA) and an ZORBAX SB C-18 column (5 μm, 9.4 × 250 mm^2^, wavelength detection at 210, 280 nm). TLC detection was performed on TLC plates (200–250 μm thickness, F254 Si gel 60, Qingdao Marine Chemical, Inc., China). The ordinary column chromatographic materials include Lichroprep RP-18 (40–63 μm, Fuji, Japan), Sephadex LH-20 (20–150 μm, Pharmacia, USA), Silical gel (200–300 mesh, Qingdao Marine Chemical, Inc., China) and Macroporous resin (0.3–1.25 mm, Mitsubishi Chemical Corporation, Japan). The industrial-grade methanol, chloroform, ethyl acetate, acetone, petroleum ether were purchased from Tianjing Chemical Reagents Co. (Tianjing, China). The analytical-grade acetonitrile were purchased from Aladdin Industrial Corporation (Shanghai, China).

### Plant Material

The air-dried cherries of *C. arabica* cultivated in Ruili of Dehong Dai and Jingpo Autonomous Prefecture (Yunnan province, China) were harvested in July 2016 and identified by Hong-bo Zhang, Dehong Institute of Tropical Agriculture. A specimen was deposited in State Key Laboratory of Phytochemistry and Plant Resources in West China, Kunming Institute of Botany, Chinese Academy of Sciences.

### Extraction and Isolation

The powder of air-dried Arabica coffee cherries (24 kg) were extracted by methanol at 80 °C for three times (3 h for each time). The methanol extract was evaporated under reduced pressure. Then, the 2.5 kg residue was suspended in water and extract with petroleum ether, ethyl acetate (EtOAc) and *n*-butanol, in turn. The EtOAc layer (200 g) was separated on a macroporous resin column (20.0 × 120 cm^2^) and eluted in a step gradient manner with MeOH/H_2_O (0:100, 20:80, 40:60, 60:40, 80:20, 100:0, v/v) to yield six fractions: Fr. A (10 g), Fr. B (24 g), Fr. C (25 g), Fr. D (38 g), Fr. E (16 g), Fr. F (30 g), respectively. Fr. E (16 g) was then further subjected to silica gel column chromatography (15.0 × 80 cm), eluting in a gradient system of CHCl_3_/MeOH (100:0 → 1:2, v/v) to yield seven sub-fractions (Fr. E-1–Fr. E-7) on the basis of TLC analysis. Fr. E-3 (2 g) was separated by use of Sephadex LH-20 (5.0 × 200 cm, eluted with MeOH, 100%, 2 L) and divided into three fractions (Fr. E-3-1–E-3-3). After that, Fr. E-3-2 (700 mg) was chromatographed on a silica column (CHCl_3_/MeOH), then separated by reverse-phase semi-preparative HPLC (CH_3_CN/H_2_O: 20 → 40%, 60 min, flow rate = 3.0 mL/min, UV, 205 nm) to get **1** (5 mg, t_*R*_ = 51.5 min) and **2** (10 mg, t_*R*_ = 46.1 min). Fr. E-4 (1.6 g) was divided into four minor fractions by use of Sephadex LH-20 (5.0 × 200 cm, eluted with MeOH, 100%, 2 L), then Fr. E-4-3 was applied to RP C-18 (3.0 × 70 cm) and eluted in a gradient of MeOH/H_2_O (40 → 80%, v/v) to yield minor fractions, then Fr. E-4-3-2 was treat by reverse-phase semi-preparative HPLC (CH_3_CN/H_2_O: 20 → 40%, 60 min, flow rate = 3.0 mL/min, UV, 205 nm) to gain **3** (9 mg, t_*R*_ = 47.3 min) and **4** (4 mg, t_*R*_ = 41.5 min).

### Spectroscopic Data of Compounds

#### Caffruone A (**1**)

White amorphous powder, $\left[ \alpha \right]_{\text{D}}^{24}$ + 1.8 (*c* = 0.09, MeOH); UV (MeOH) *λ*_max_ (log *ε*): 203 (3.85), 372 (2.97) nm; IR (KBr) *v*_max_: 3431, 3080, 2975, 2872, 1745, 1690, 1620, 1460, 1380, 1000, 906 cm^−1^; HRESIMS *m*/*z* 513.3551 [M + Na]^+^ (calcd for C_30_H_50_O_5_ Na, 513.3550); ^1^H and ^13^C NMR data shown in Tables 1 and 2.

#### Caffruone B (**2**)

White amorphous powder, $\left[ \alpha \right]_{\text{D}}^{24}$ – 4.8 (*c* = 0.2, MeOH); UV (MeOH) *λ*_max_ (log *ε*): 202 (3.77), 268 (2.86), 374 (0.0067) nm; IR (KBr) *v*_max_: 3430, 3083, 2965, 2872, 1680, 1610, 1460, 1380, 1010, 900 cm^−1^; HRESIMS *m*/*z* 513.3551 [M + Na]^+^ (calcd for C_30_H_50_O_5_ Na, 513.3550); ^1^H and ^13^C NMR data shown in Tables 1 and 2.

#### Caffruone C (**3**)

White amorphous powder, $\left[ \alpha \right]_{\text{D}}^{24}$ – 1.9 (*c* = 0.2, MeOH); UV (MeOH) *λ*_max_ (log *ε*): 203 (3.36), 278 (2.39) nm; IR (KBr) *v*_max_: 3410, 3080, 2960, 2870, 1724, 1685, 1615, 1460, 1380, 1020, 905 cm^−1^; HRESIMS *m*/*z* 511.3396 [M + Na]^+^ (calcd for C_30_H_48_O_5_ Na, 511.3394); ^1^H and ^13^C NMR data shown in Tables 1 and 2.

#### Caffruone D (**4**)

White amorphous powder, $\left[ \alpha \right]_{\text{D}}^{24}$ + 18.9 (*c* = 0.1, MeOH); UV (MeOH) *λ*_max_ (log *ε*): 203 (3.89), 269 (2.83) nm; IR (KBr) *v*_max_: 3420, 3080, 2970, 2880, 1690, 1610, 1460, 1380, 1020, 920 cm^−1^; HRESIMS *m*/*z* 511.3397 [M + Na]^+^ (calcd for C_30_H_48_O_5_ Na, 511.3394); ^1^H and ^13^C NMR data shown in Tables 1 and 2.

## Electronic supplementary material

Below is the link to the electronic supplementary material.

Supplementary material 1 (DOCX 3207 kb)
