# Supplementary material for: New Dammarane Triterpenoids, Caffruones A–D, from the Cherries of Coffea arabica
Source: Nat Prod Bioprospect. 2018 Aug 20;8(6):413–8. doi: 10.1007/s13659-018-0181-y (PMC6224810; doi:10.1007/s13659-018-0181-y)
Supplement: Supplementary file 1 — Supplementary material 1 (DOCX 3207 kb) [file 13659_2018_181_MOESM1_ESM.docx]

**Fig 1.** The ^1^H NMRspectrum of compound **1**.


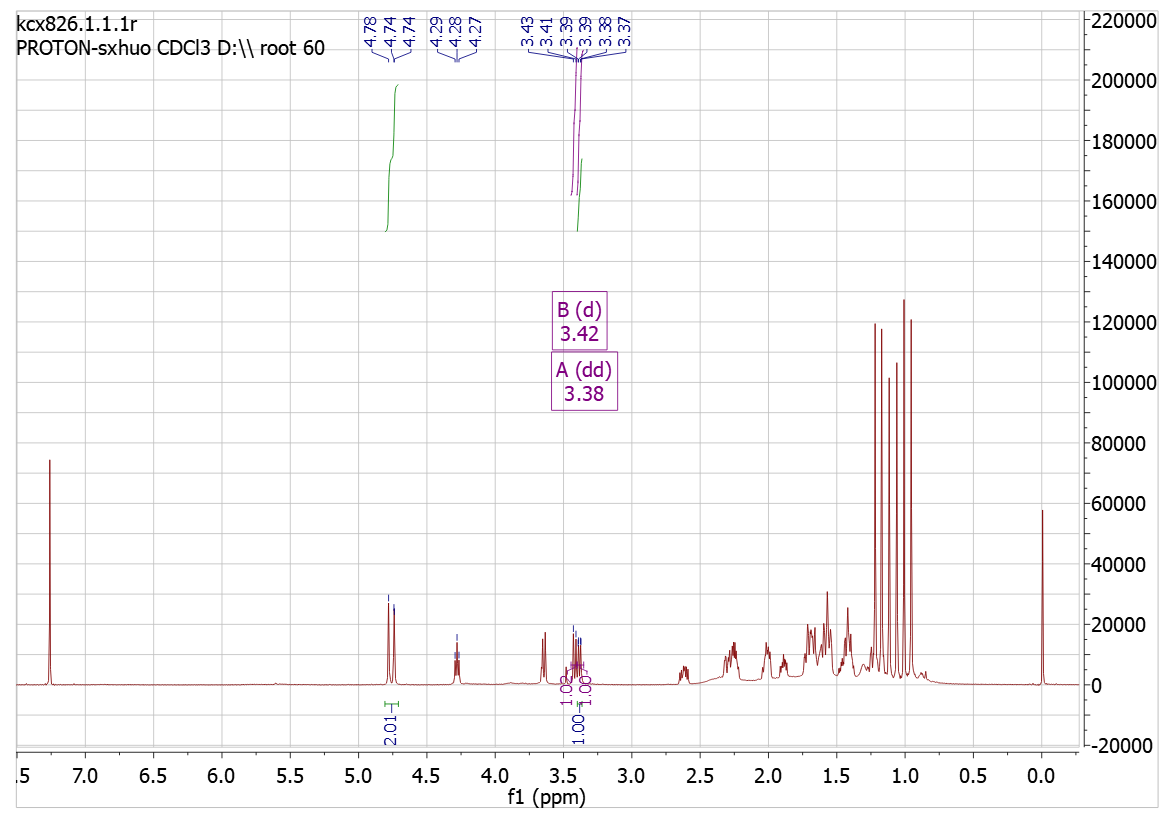


**Fig 2.** The ^13^C NMR spectrum of compound **1**.


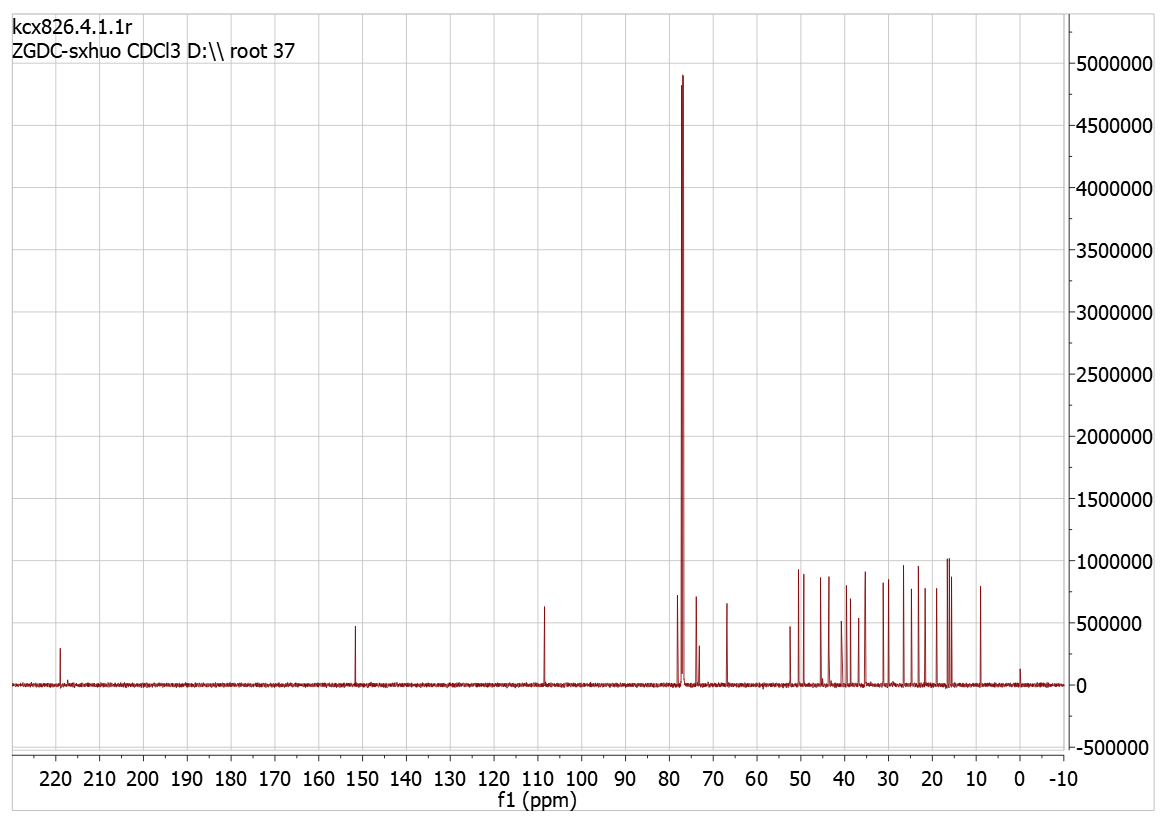


**Fig 3.** The HSQC correlations of compound **1**.


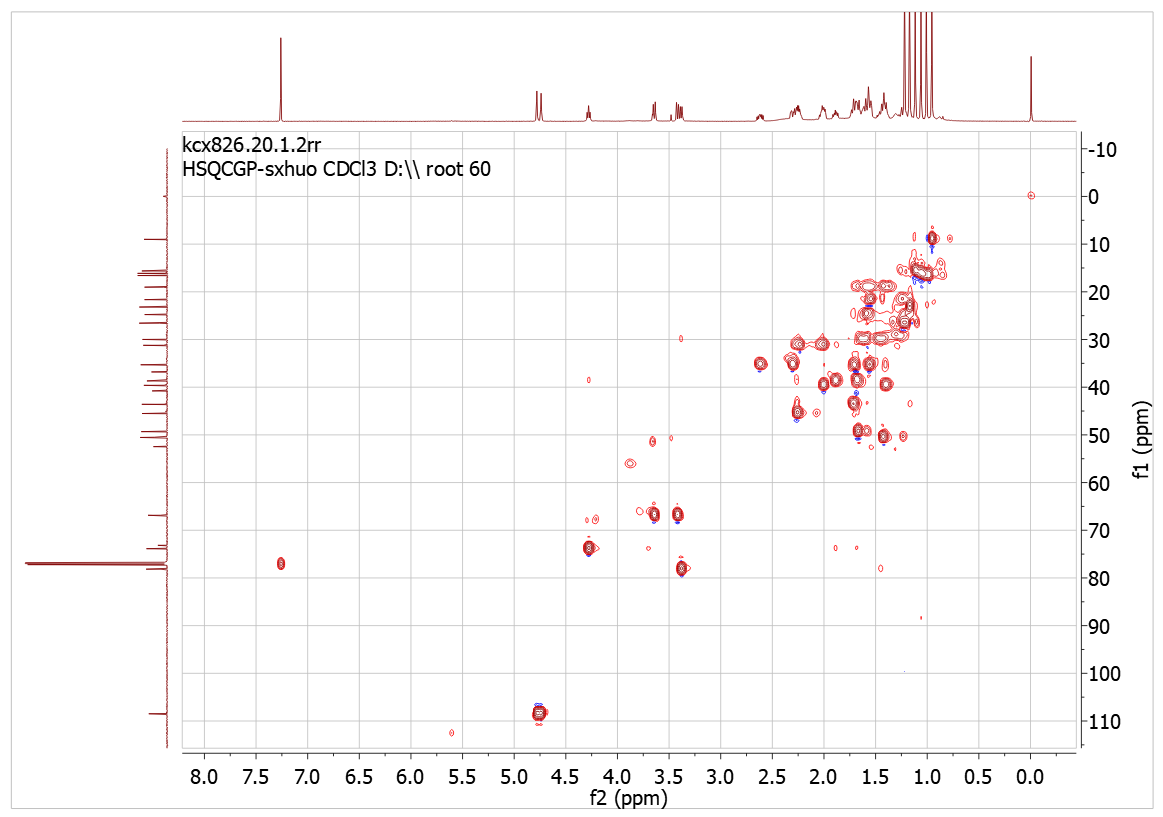


**Fig 4.** The HMBC correlations of compound **1**.


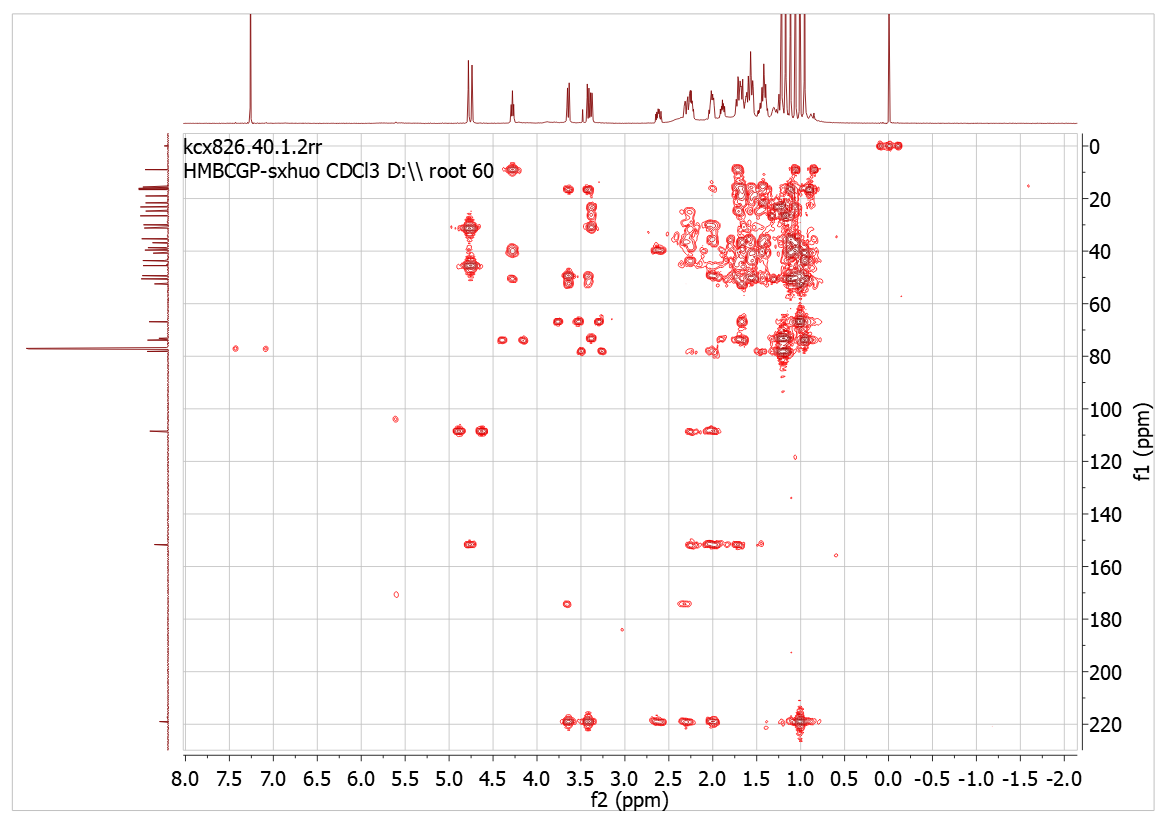


**Fig 5.** The ^1^H-^1^H COSYcorrelations of compound **1**.


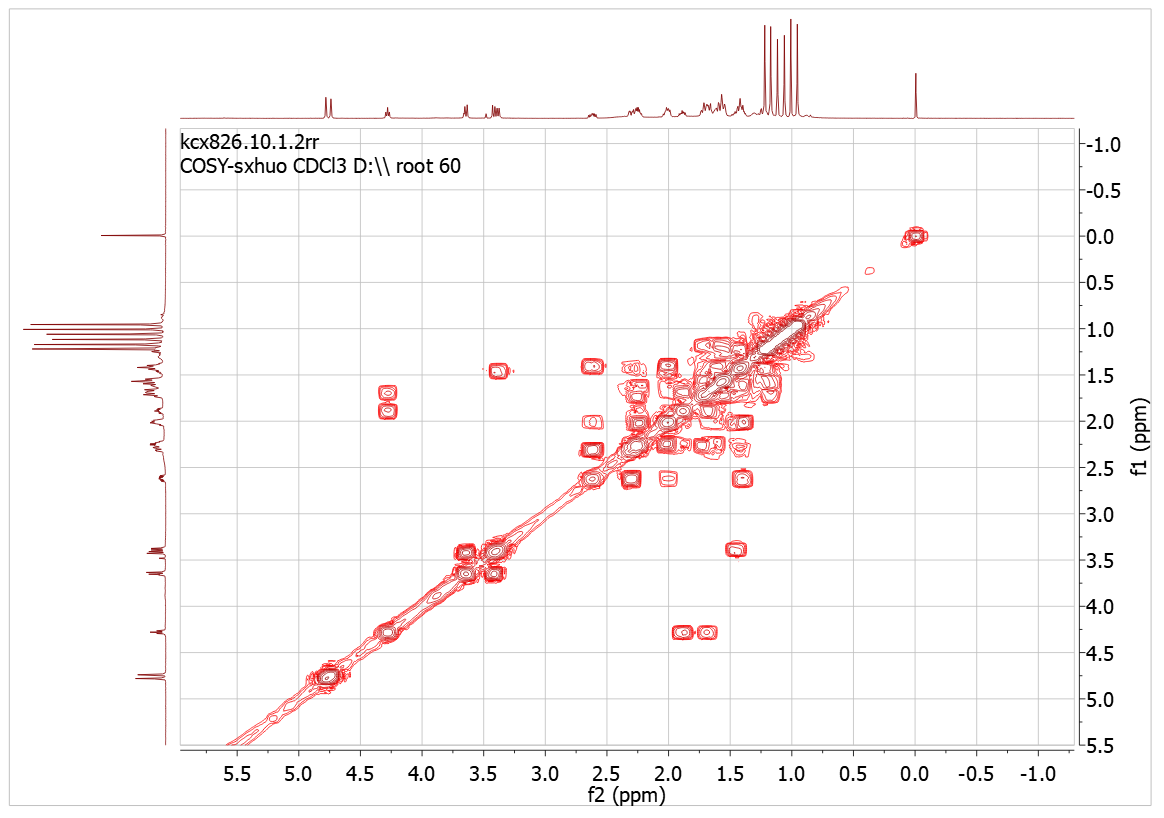


**Fig 6.** The ROESYcorrelations of compound **1**.


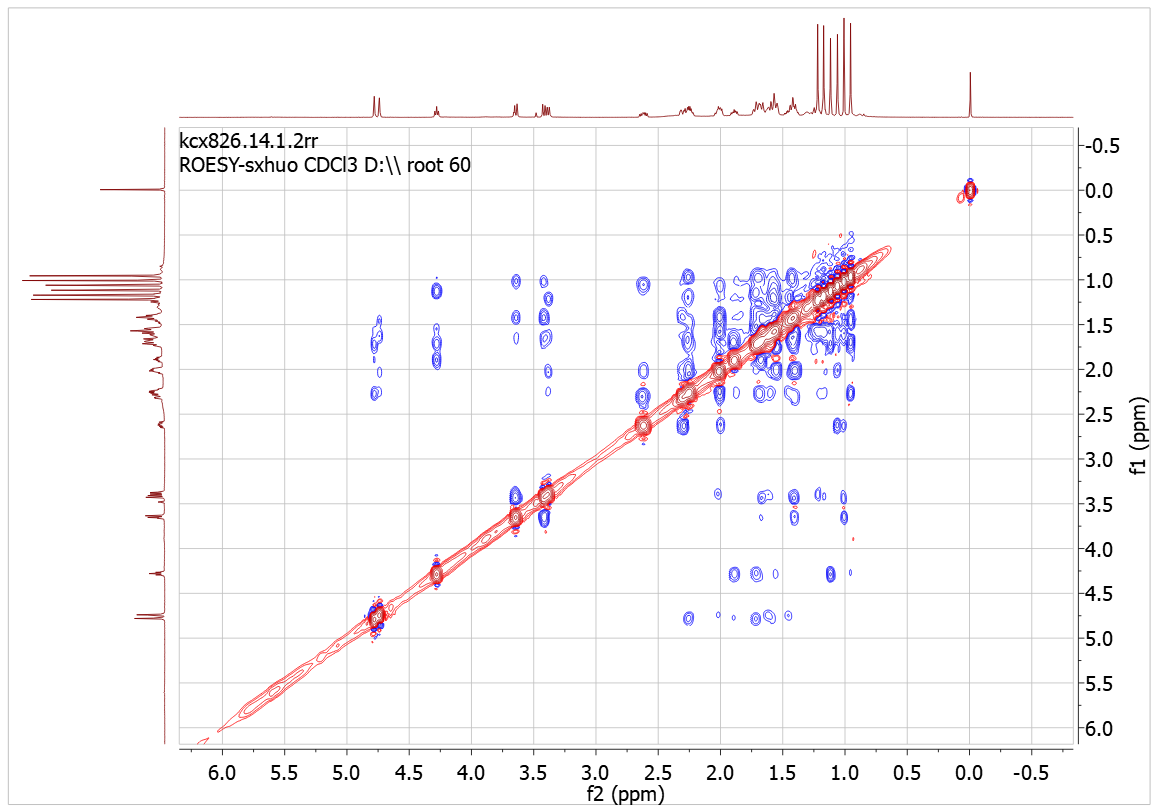


**Fig 7.** The ^1^H NMRspectrum of compound **2**.

**
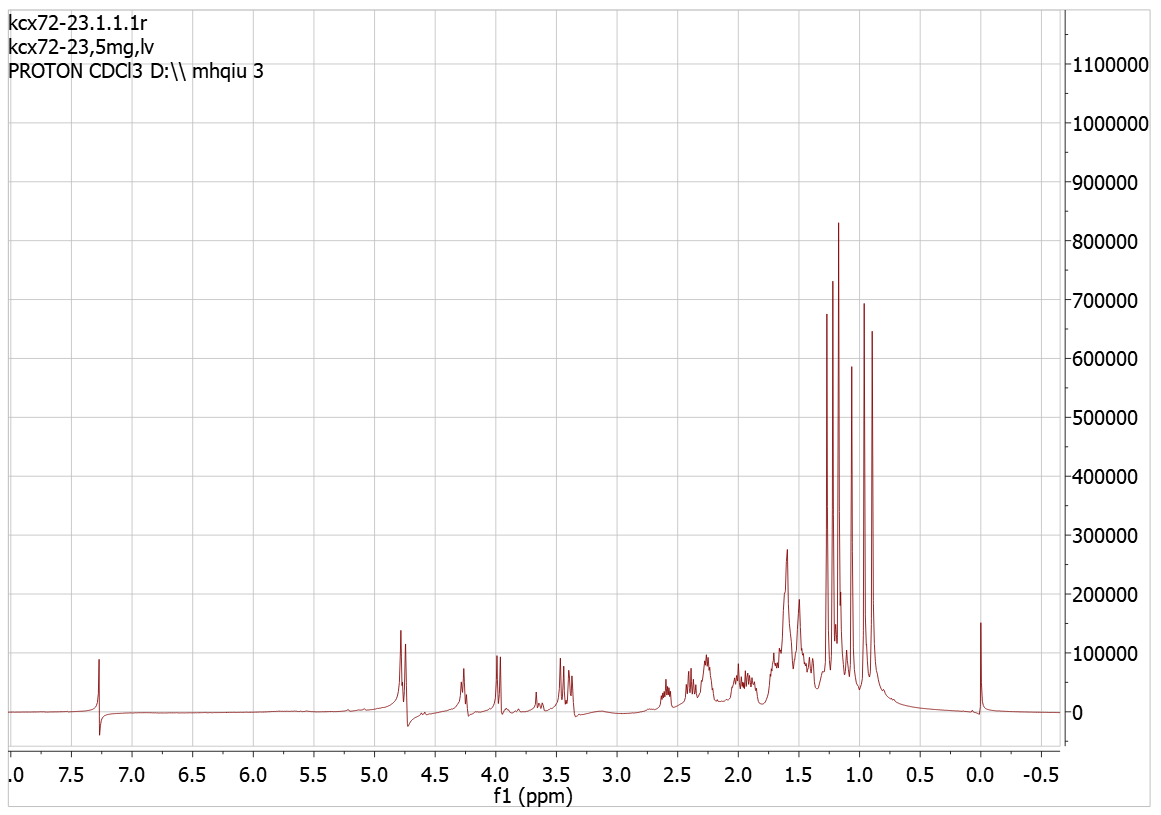
**

**Fig 8.** The ^13^C NMR spectrum of compound **2**.

**
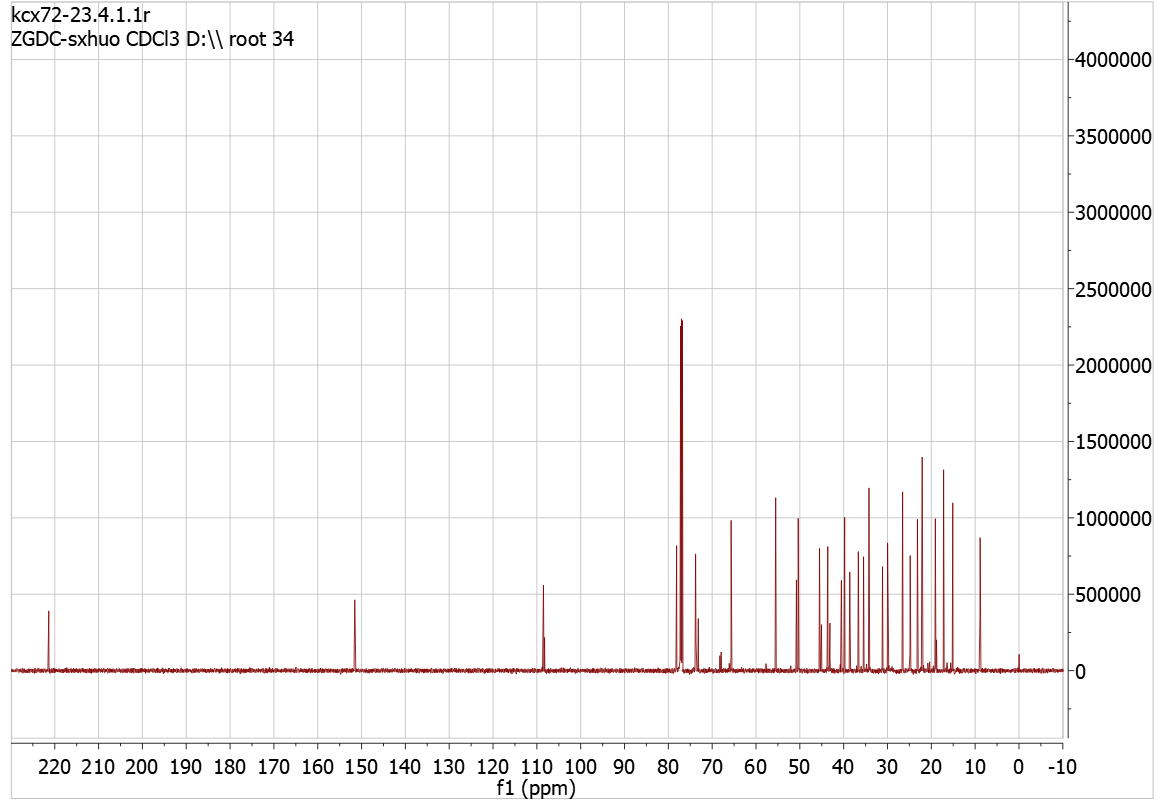
**

**Fig 9.** The HSQC correlations of compound **2**.

**
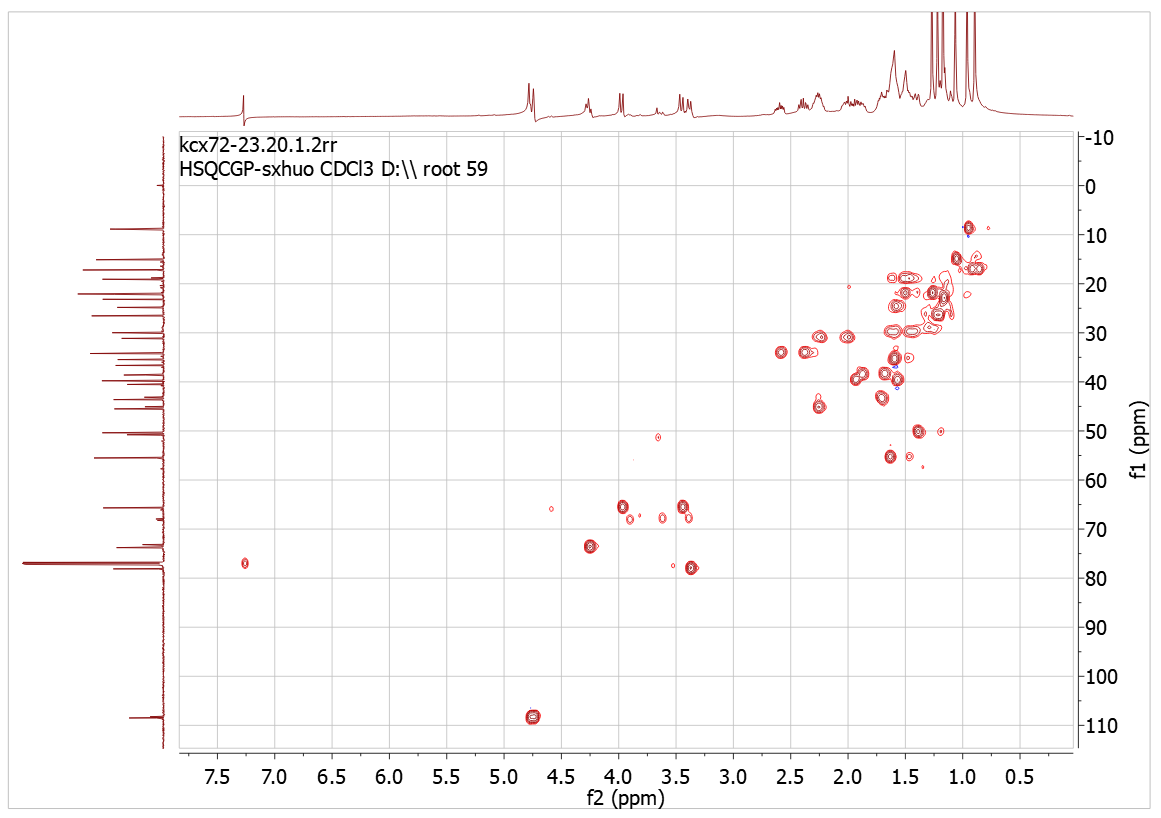
**

**Fig 10.** The HMBC correlations of compound **2**.

**
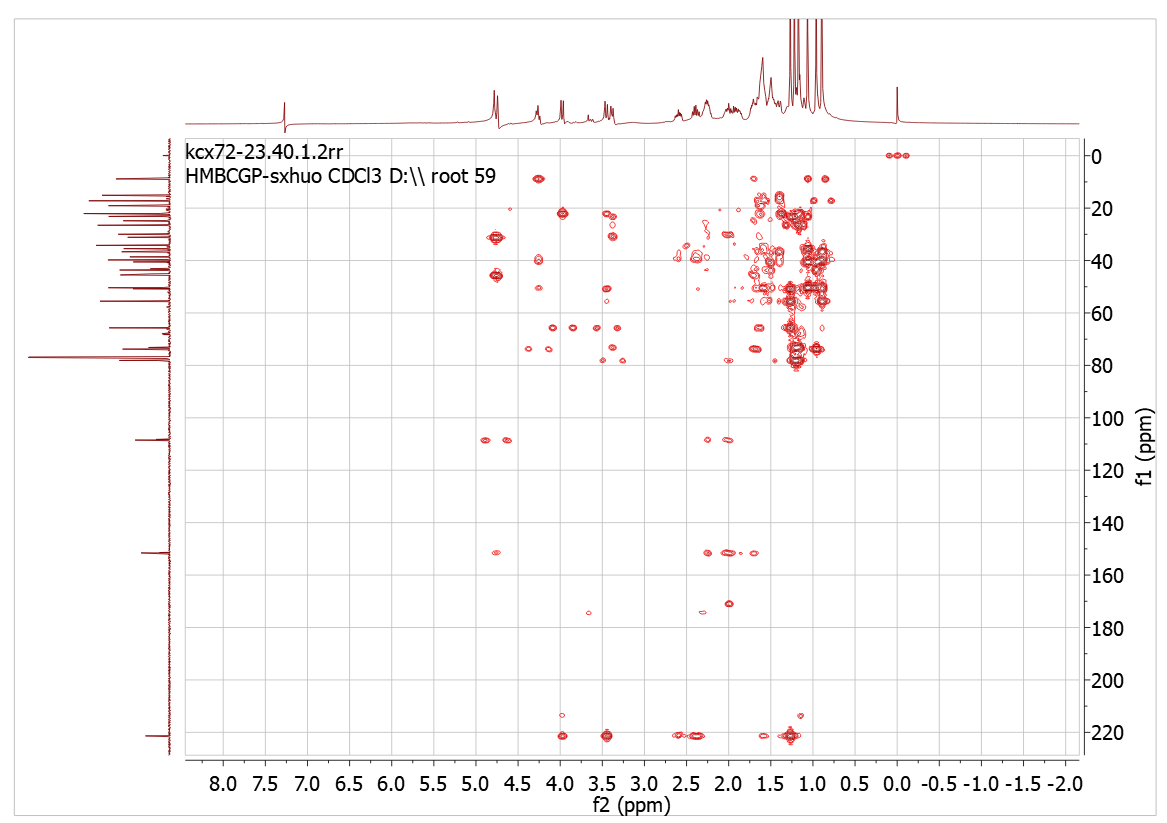
**

**Fig 11.** The ^1^H-^1^H COSYcorrelations of compound **2**.

**
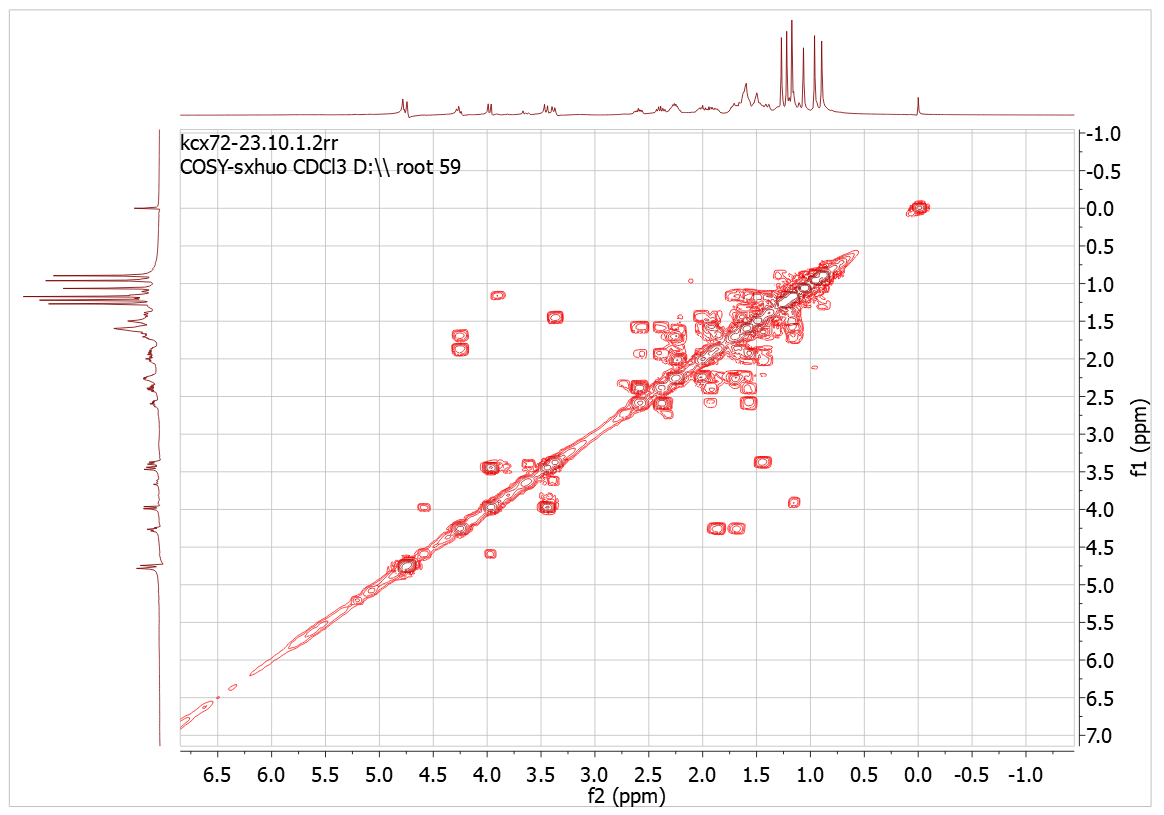
**

**Fig 12.** The ROESYcorrelations of compound **2**.

**
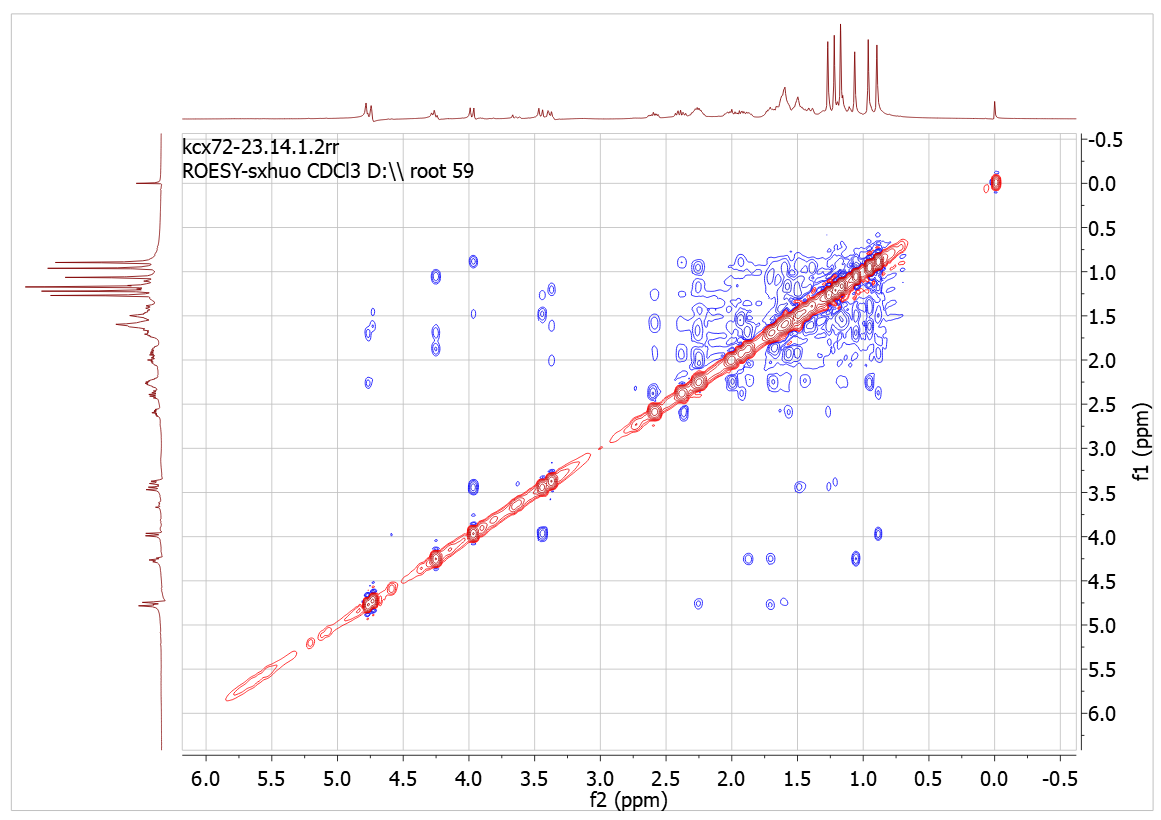
**

**Fig 13.** The ^1^H NMRspectrum of compound **3**.

**
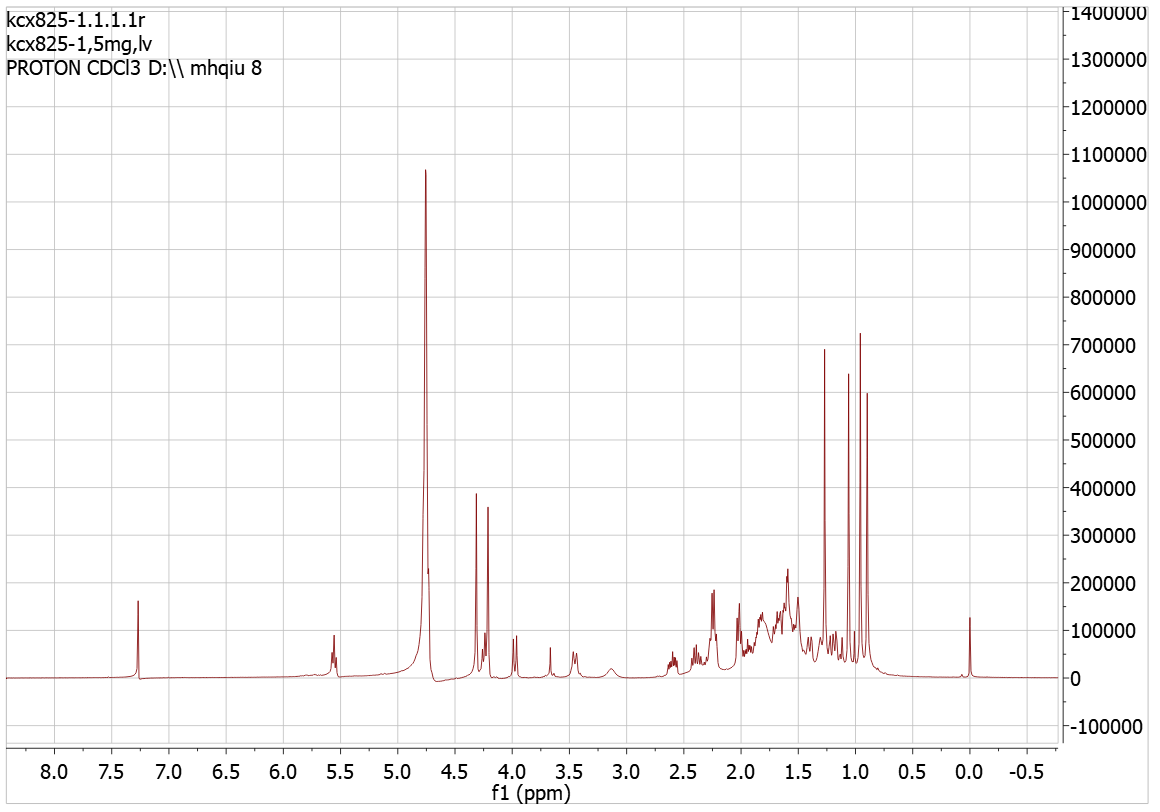
**

**Fig 14.** The ^13^C NMR spectrum of compound **3**.

**
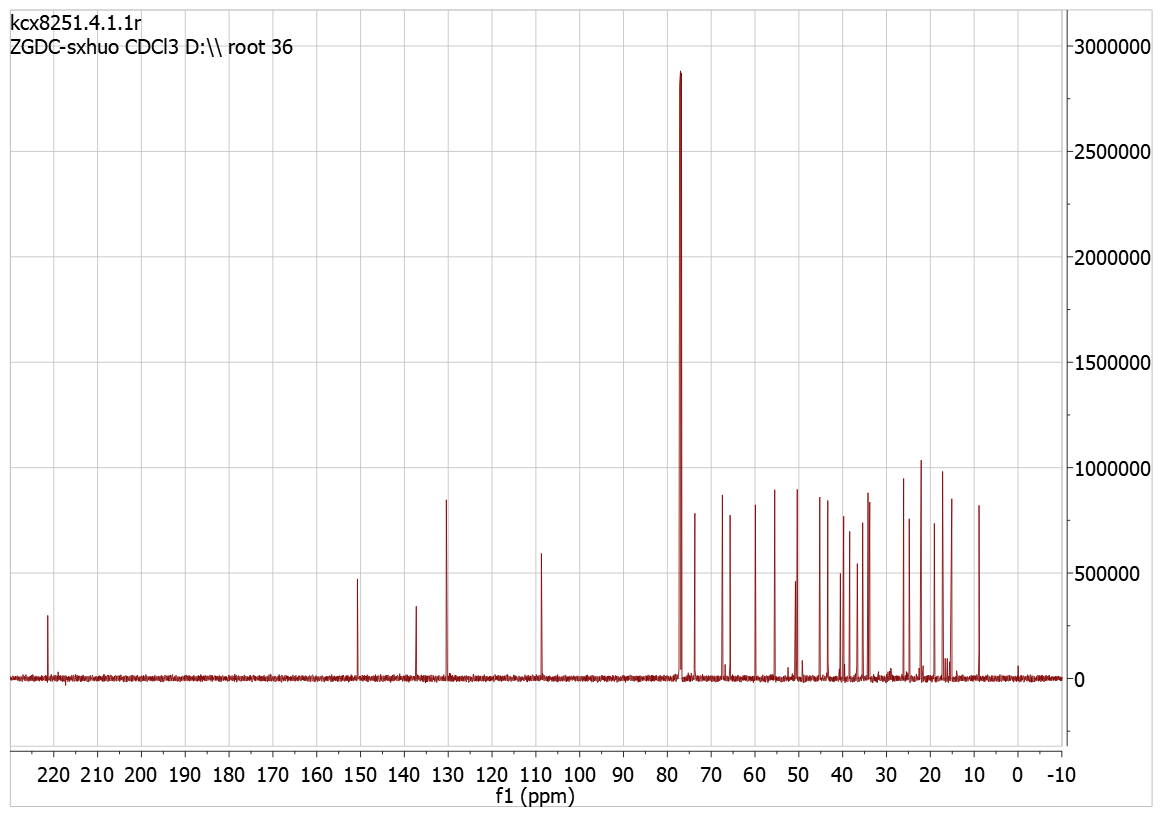
**

**Fig 15.** The HSQC correlations of compound **3**.

**
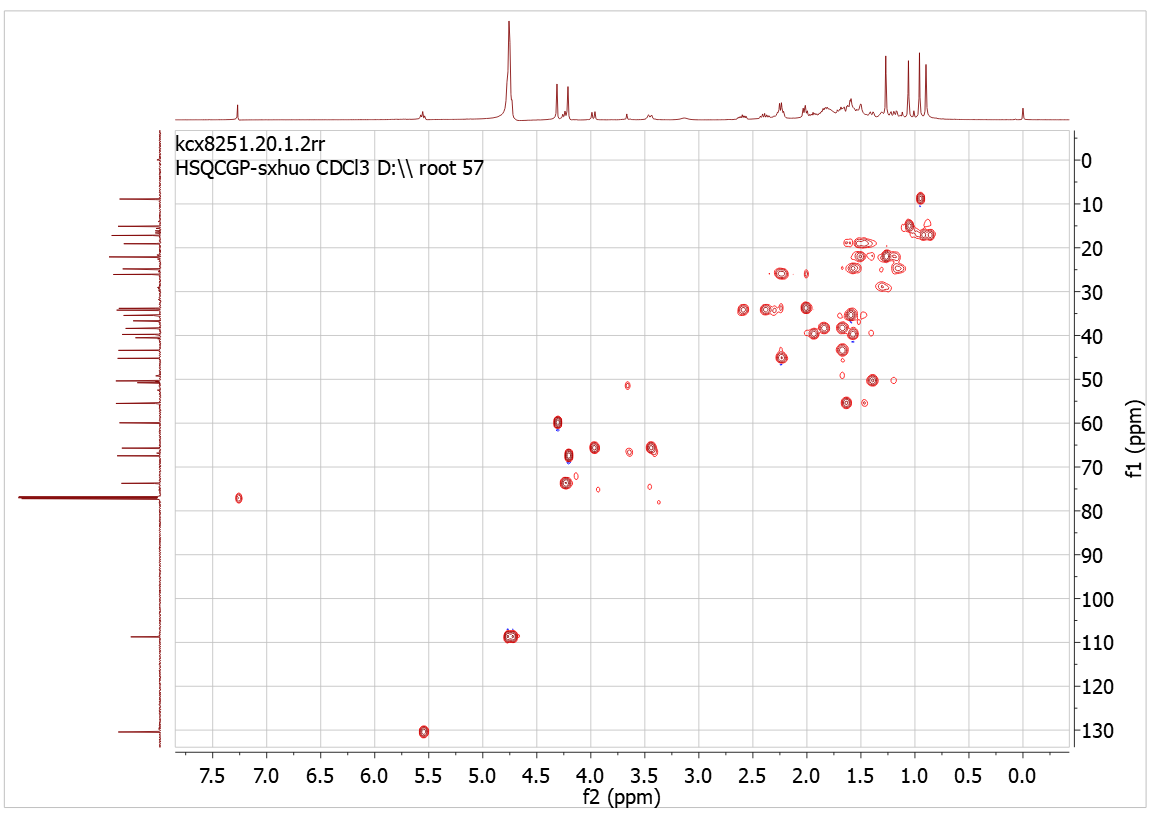
**

**Fig 16.** The HMBC correlations of compound **3**.

**
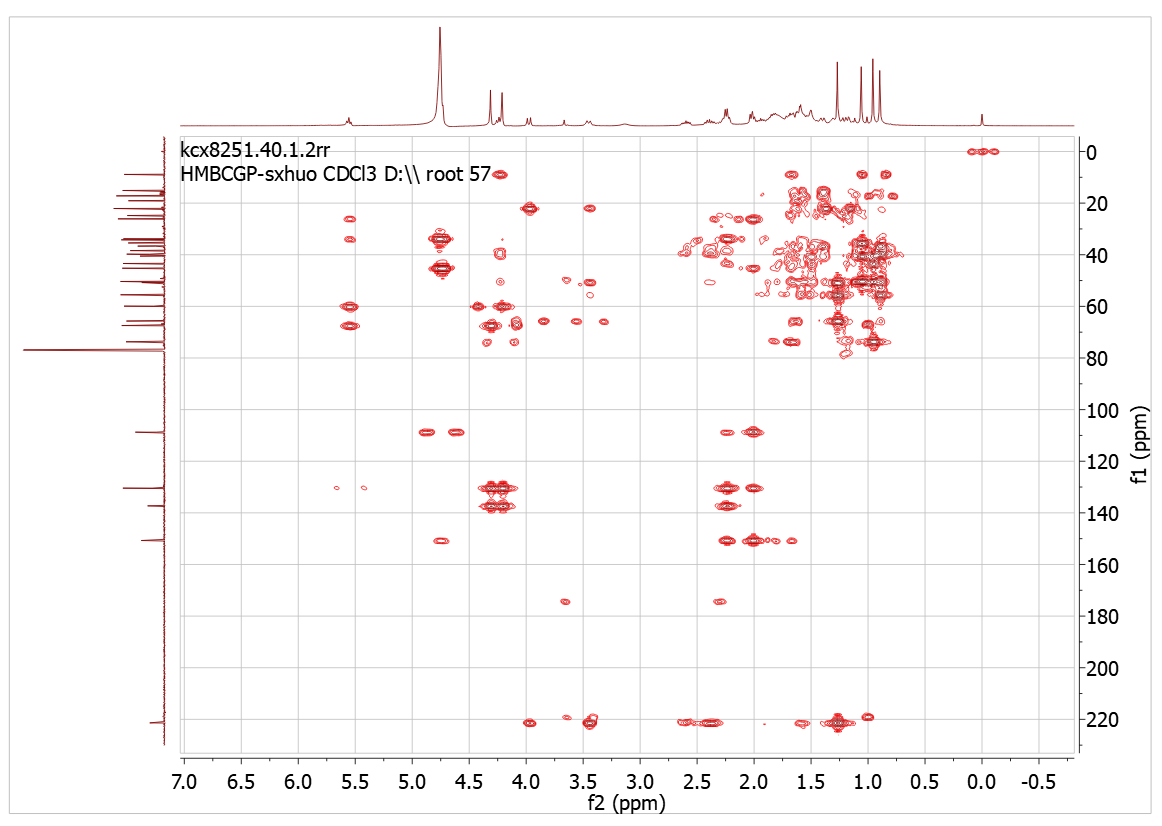
**

**Fig 17.** The ^1^H-^1^H COSYcorrelations of compound **3**.

**
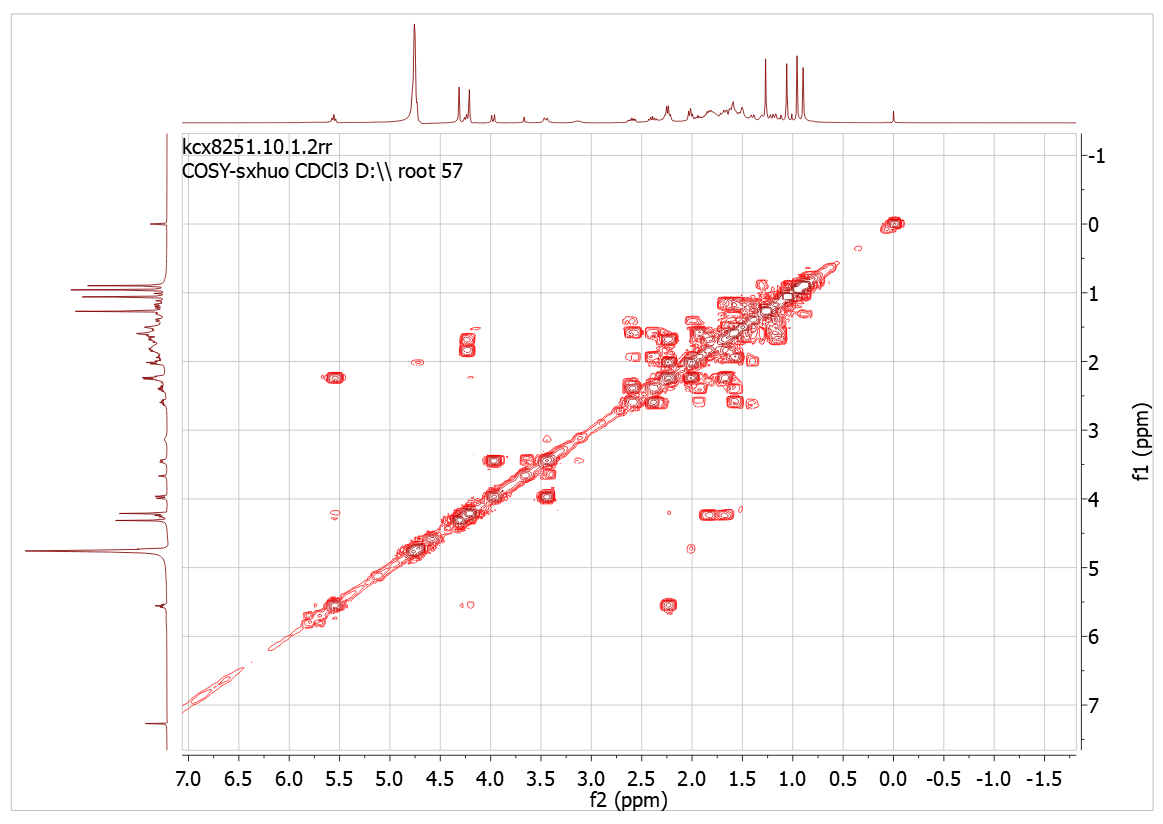
**

**Fig 18.** The ROESYcorrelations of compound **3**.

**
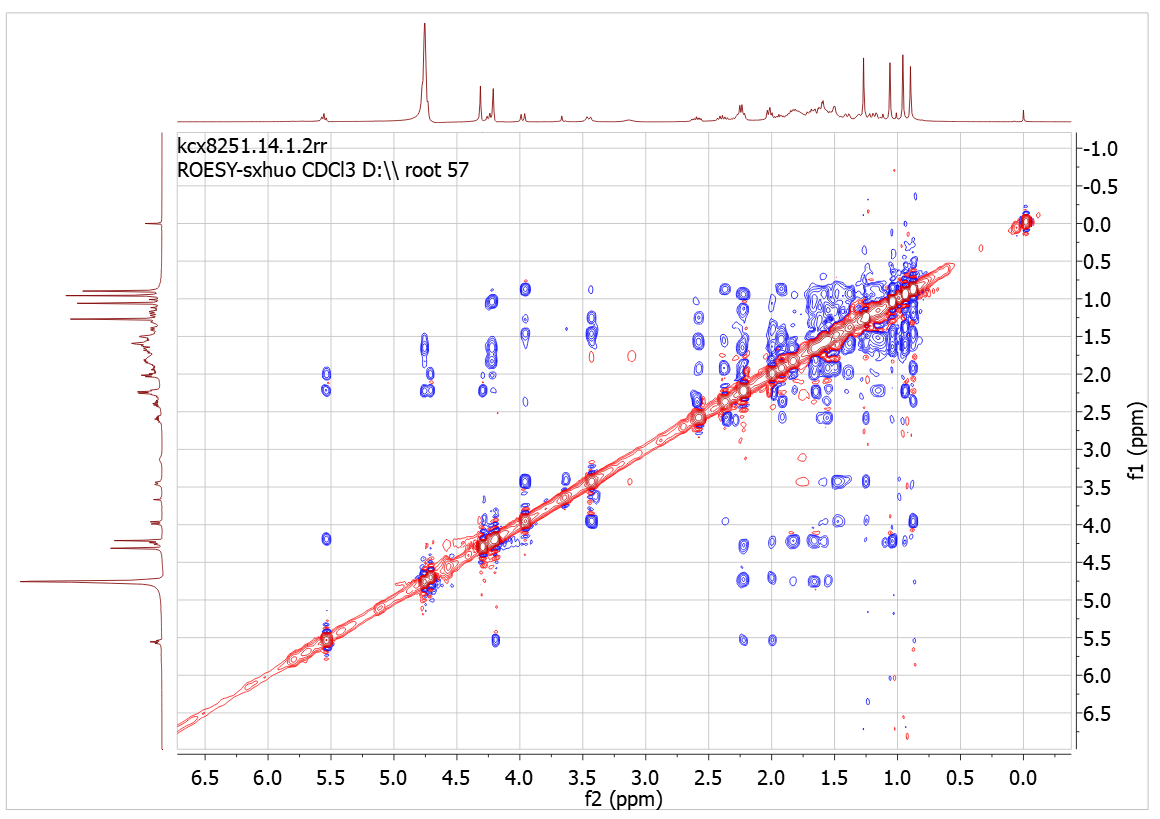
**

**Fig 19.** The ^1^H NMRspectrum of compound **4**.


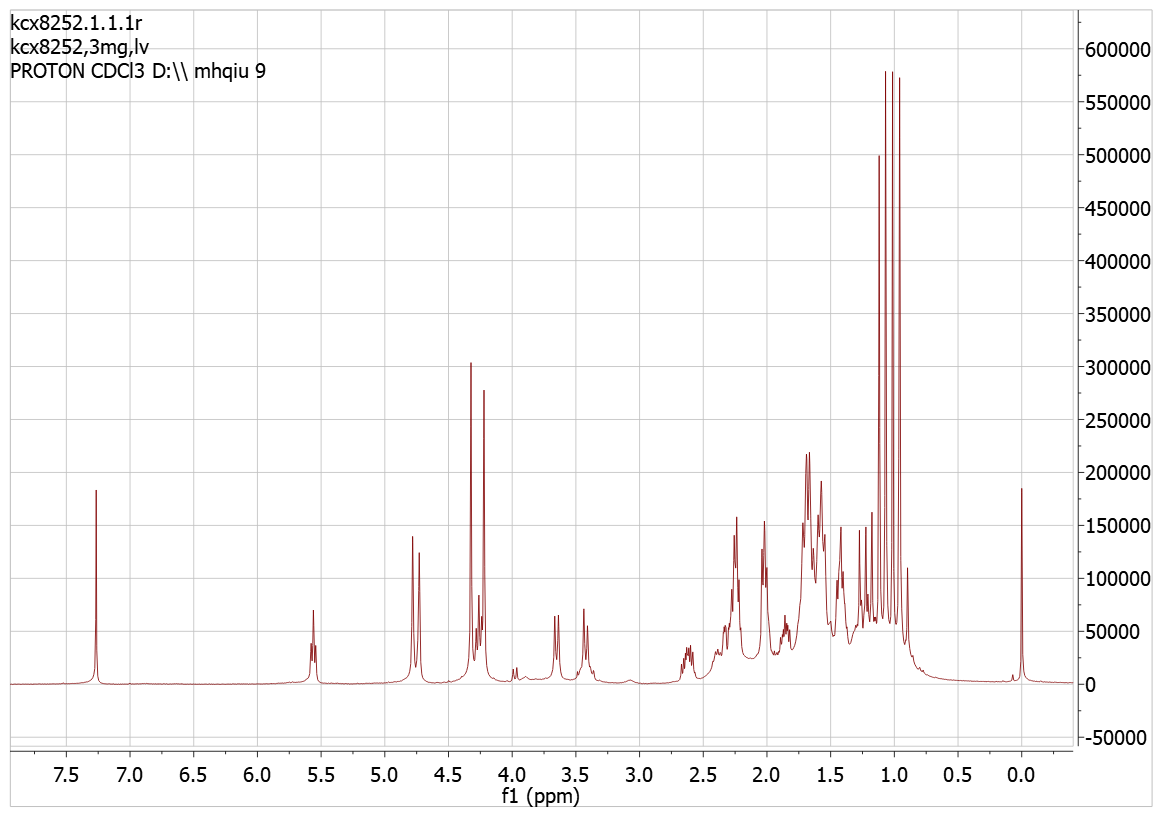


**Fig 20.** The ^13^C NMR spectrum of compound **4**.


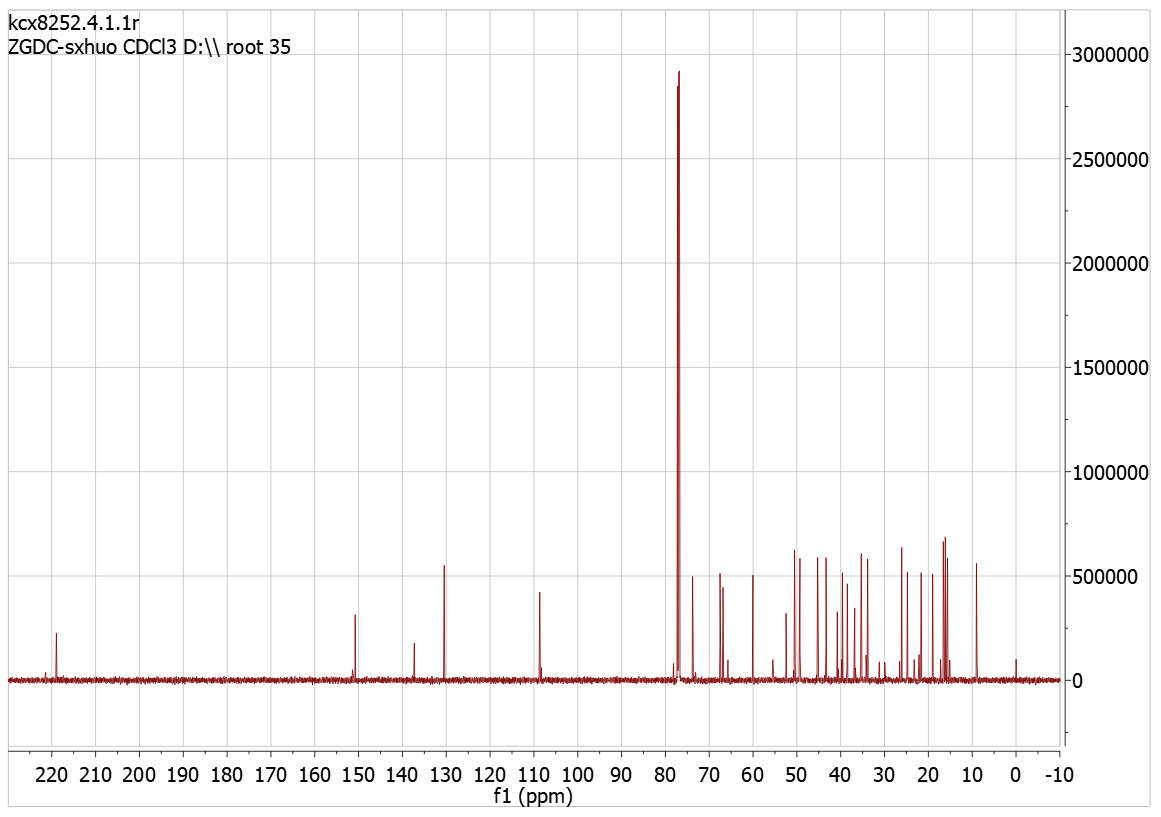


**Fig 21.** The HSQC correlations of compound **4**.


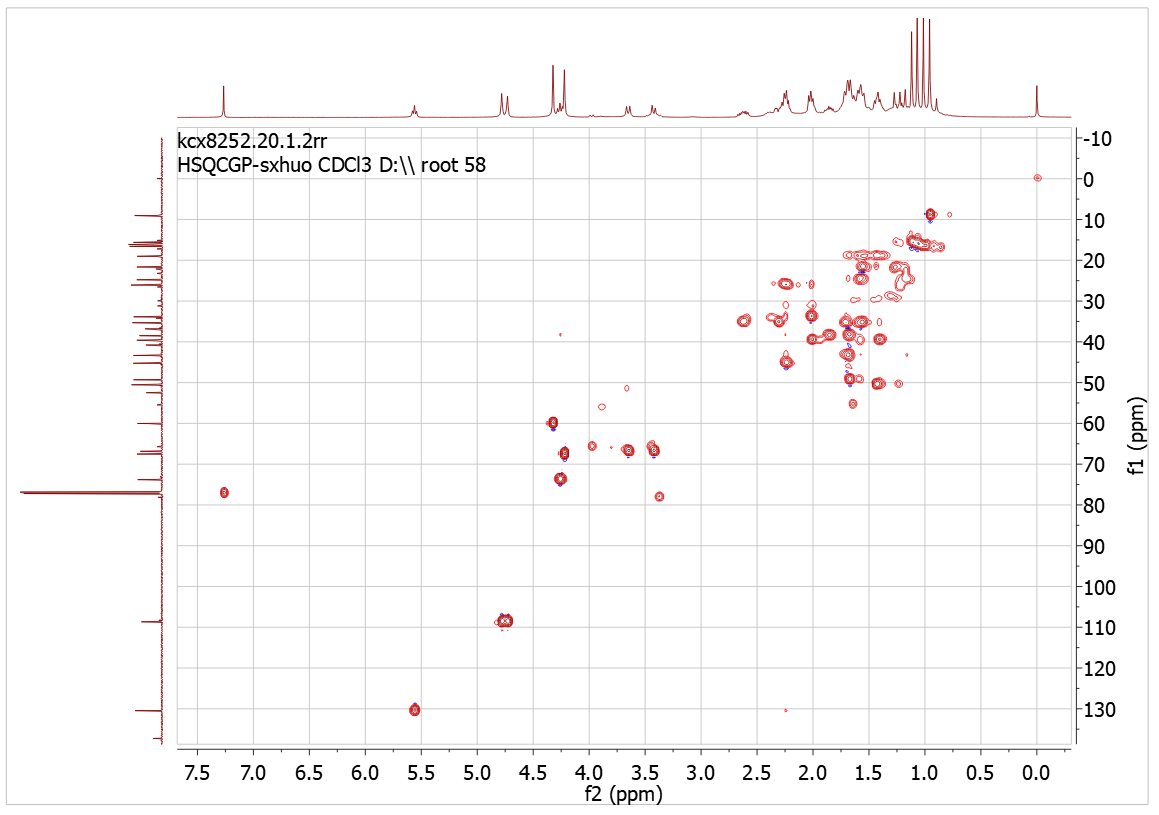


**Fig 22.** The HMBC correlations of compound **4**.


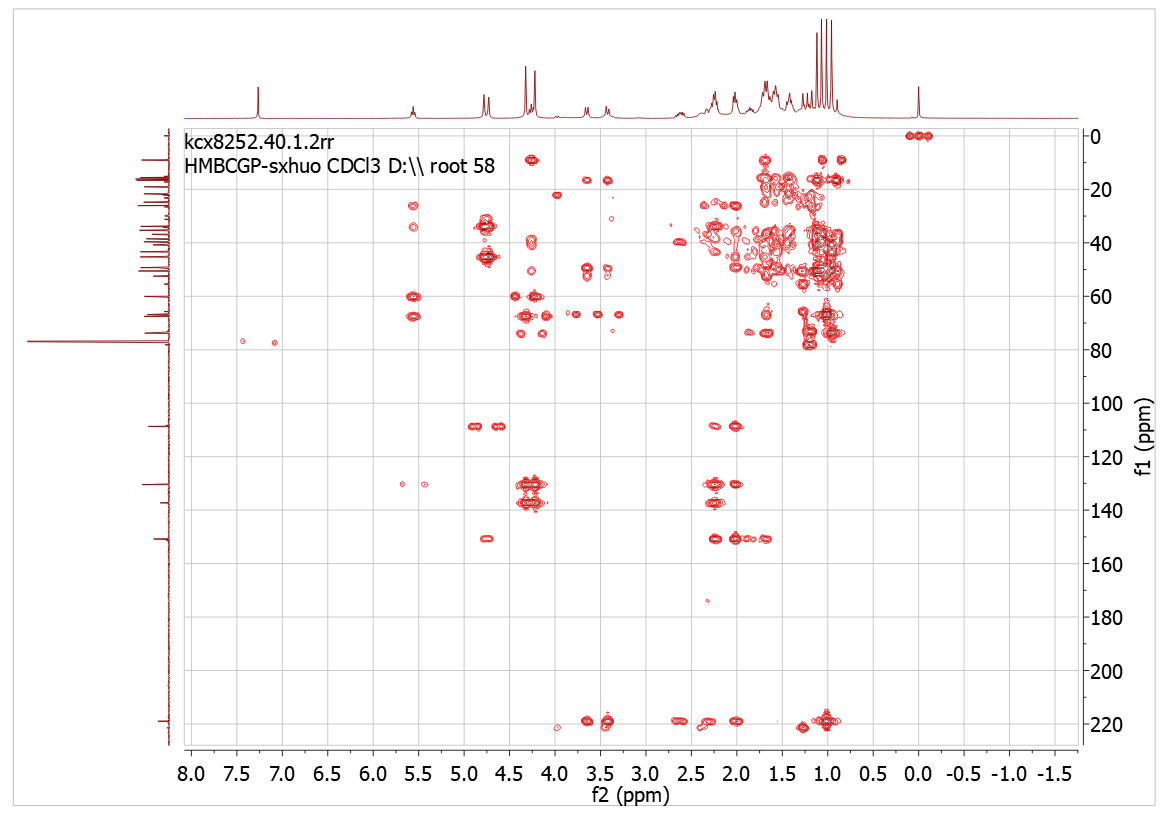


**Fig 23.** The ^1^H-^1^H COSYcorrelations of compound **4**.


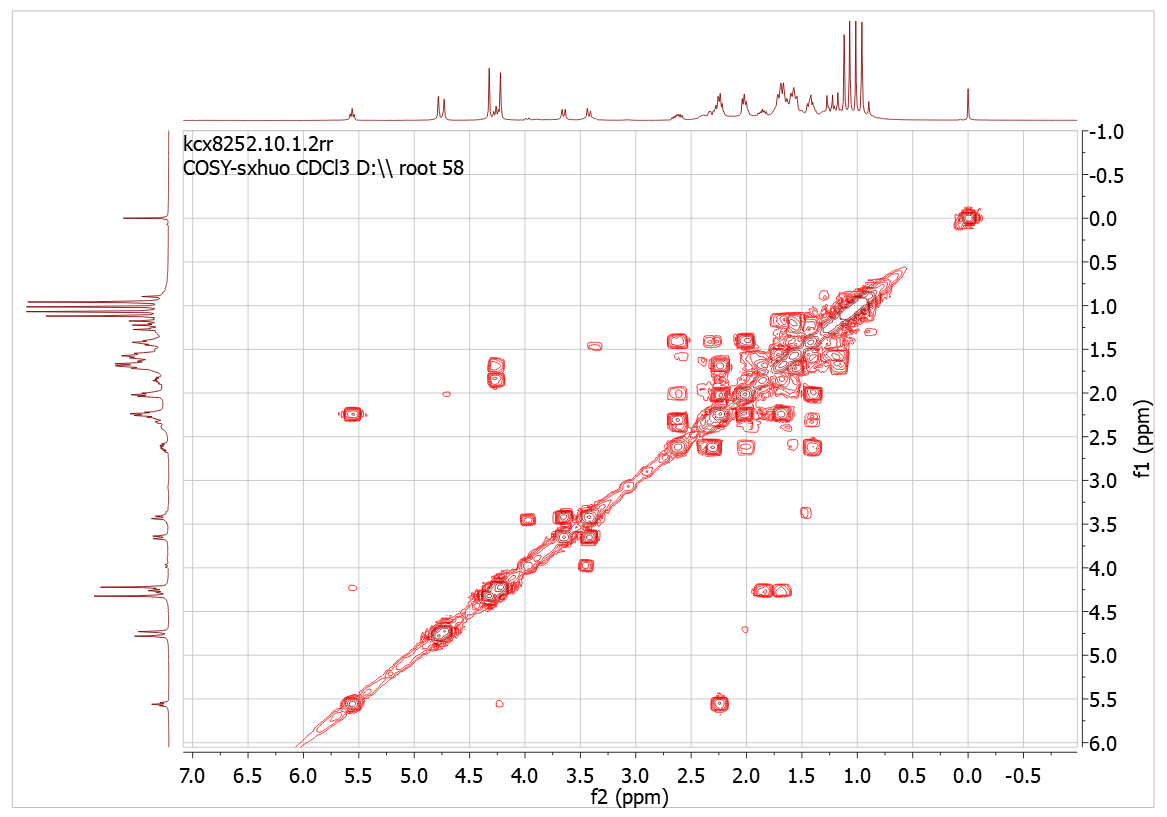


**Fig 24.** The ROESYcorrelations of compound **4**.


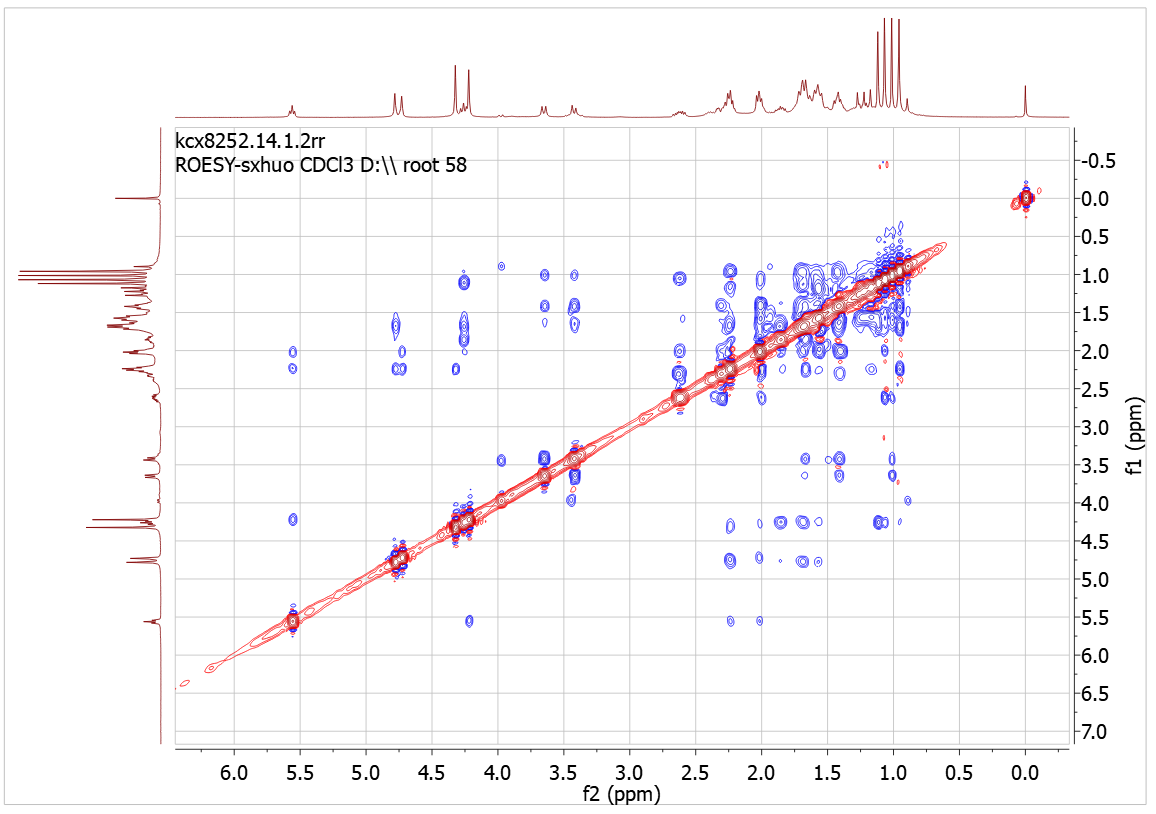

**Fig 25.** The key HMBC, ROESY and ^1^H-^1^H COSY correlations of compound **2**.

**Fig 26.** The key HMBC and ROESY correlations of compound **4**.
